# Aerobic capacity over 16 years in patients with rheumatoid arthritis: Relationship to disease activity and risk factors for cardiovascular disease

**DOI:** 10.1371/journal.pone.0190211

**Published:** 2017-12-22

**Authors:** Kristina Hörnberg, Björn Sundström, Lena Innala, Solbritt Rantapää-Dahlqvist, Solveig Wållberg-Jonsson

**Affiliations:** Department of Public Health and Clinical Medicine/Rheumatology, University of Umeå, Umeå, Sweden; Medical University Innsbruck, AUSTRIA

## Abstract

The aim of this study was to analyse the change in aerobic capacity from disease onset of rheumatoid arthritis (RA) over 16.2 years, and its associations with disease activity and cardiovascular risk factors. Twenty-five patients (20 f/5 m), diagnosed with RA 1995-2002 were tested at disease onset and after mean 16.2 years. Parameters measured were: sub-maximal ergometer test for aerobic capacity, functional ability, self-efficacy, ESR, CRP and DAS28. At follow-up, cardiovascular risk factors were assessed as blood lipids, glucose concentrations, waist circumference, body mass index (BMI), body composition, pulse wave analysis and carotid intima-media thickness. Aerobic capacity [median (IQR)] was 32.3 (27.9-42.1) ml O_2_/kg x min at disease onset, and 33.2 (28.4-38.9) at follow-up (p>0.05). Baseline aerobic capacity was associated with follow-up values of: BMI (r_s_ = -.401, p = .047), waist circumference (r_s_ = -.498, p = .011), peripheral pulse pressure (r_s_ = -.415, p = .039) self-efficacy (r_s_ = .420, p = .037) and aerobic capacity (r_s_ = .557, p = .004). In multiple regression models adjusted for baseline aerobic capacity, disease activity at baseline and over time predicted aerobic capacity at follow-up (AUC DAS28, 0-24 months; **β** = -.14, p = .004). At follow-up, aerobic capacity was inversely associated with blood glucose levels (r_s_ = -.508, p = .016), BMI (r_s_ = -.434, p = .030), body fat% (r_s_ = -.419, p = .037), aortic pulse pressure (r_s_ = -.405, p = .044), resting heart rate (r_s_ = -.424, p = .034) and self-efficacy (r_s_ = .464, p = .020) at follow-up. We conclude that the aerobic capacity was maintained over 16 years. High baseline aerobic capacity associated with favourable measures of cardiovascular risk factors at follow-up. Higher disease activity in early stages of RA predicted lower aerobic capacity after 16.2 years.

## Introduction

Patients with rheumatoid arthritis (RA) suffer from increased mortality due to cardiovascular disease (CVD) [1, 2]. Traditional risk factors, *e*.*g*., smoking, hypertension, dyslipidaemia, insulin resistance and body fat, contribute to the increased risk for CVD [3]. However, the chronic systemic inflammation in RA is involved in the progression of atherosclerosis [4] thereby increasing the risk of suffering cardiovascular events.

Aerobic capacity (VO_2_ max) is inversely, and strongly, related to CVD, and all-cause mortality in apparently healthy and in patients with CVD [5, 6]. As a matter of fact, aerobic capacity has been described as a more powerful predictor for CVD than traditional risk factors [7]. Studies describing the impact of aerobic capacity on cardiovascular health in patients with RA are scarce, but Metsios *et al*. [8], found a deteriorated cardiovascular profile and increased 10-year cardiovascular risk in patients with lower aerobic capacity. Furthermore, exercise has been shown to positively affect the cardiovascular profile in patients with RA [9–12].

Thus, there is reason to believe that greater aerobic capacity may provide a protection against CVD in patients with RA. In a previous study from 2007, we concluded that the aerobic capacity of patients with early RA did not change over the first two years [13]. However, prospective studies analysing the changes in aerobic capacity from disease onset and over a longer period of time, are lacking. There is also a need to learn more about factors predicting aerobic capacity in order to prevent its deterioration. Self-efficacy, which is explained as the confidence in one´s ability to perform a given behaviour despite obstacles [14], has been described as an important predictor for sustained physical activity and exercise in patients with RA [15].

The aim of the present study was (*i*) to analyse aerobic capacity at disease onset and after 16 years and the change over time in patients with RA, and (*ii*) to describe factors associated with aerobic capacity at follow-up. We also aimed (*iii*) to identify possible associations between baseline aerobic capacity and future measures of CVD risk factors and atherosclerosis. Finally, *(iv)*, we wished to identify baseline factors that predict aerobic capacity after 16 years.

## Patients and methods

### Patients

Since 1995 all individuals with early RA (*i*.*e*., symptomatic for <12 months), fulfilling the American College of Rheumatology criteria for RA [16], were included in the early arthritis cohort, and followed at the Department of Rheumatology in Umeå, county of Västerbotten, Sweden. Over the years, measures of disease activity, body function and functional ability were recorded regularly. A sub maximal test for aerobic capacity was performed, unless they were aged > 65 years, prescribed beta blocking agents or had physical impairment, hindering the test. Following years, patients were offered medical and rehabilitative care, based on individual needs. Medical treatment followed the standard care aiming at remission. The objective of rehabilitation was to minimize impairment and disability by providing information about how to manage different consequences of the disease and by encouraging physical activity.

**Fig 1:**
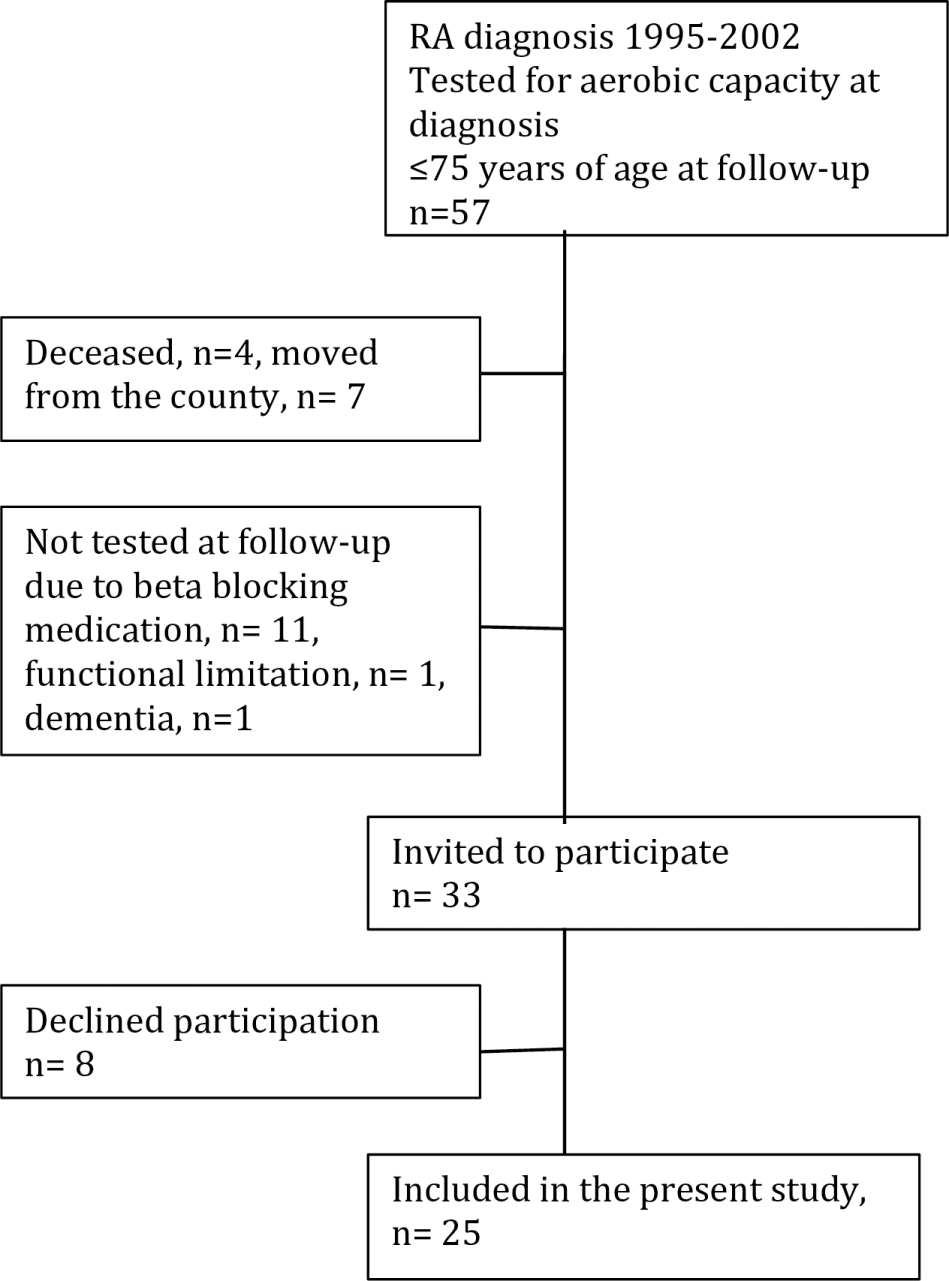
Flowchart over inclusion and exclusion process.

Patients eligible to participate in the present follow-up study were those incorporated into the RA cohort between 1995 and 2002, who performed tests for aerobic capacity at the time of diagnosis, and were aged ≤75 years at follow-up (n = 57) (Fig 1).

Over the years, four of these 57 patients had died (cancer (n = 3); ischaemic heart disease (n = 1); and seven had moved out of the area. At the time of follow-up, thirteen patients were excluded due to: beta blocking medication initiated after the baseline investigation (n = 11), physical impairment (n = 1) and dementia (n = 1). In total, 33 patients (26 women and 7 men) were invited to participate in the study, of whom 25 (20 women and 5 men) accepted. All patients gave their informed consent after receiving verbal and written information. The study was approved by the Ethical Committee of the University in Umeå, Sweden (Dnr 2014/356-31) and was performed in accordance with the Helsinki Declaration.

### Baseline measures

Disease activity was assessed at the time of diagnosis, after twelve and 24 months, using Disease Activity Score (DAS28) [17], comprising the composite of the number of tender and swollen joints, patient self-assessed general health, and the erythrocyte sedimentation rate (ESR). The area under the curve (AUC) for DAS 28 was calculated from the baseline values recorded during the preceding 24 months. C-reactive protein (CRP, mg/L), glucose (mmol/L) and creatinine (mmol/L) were analysed according to routine methods. Rheumatoid factor (RF) was analysed using Waaler Rose (a titer of 1/80 corresponds to a positive value), and anti-CCP antibody (ACPA) using enzyme-linked immunoassays (ELISA) for anti-CCP antibodies type 2. Plasma levels of cholesterol, triglycerides and HDL were analysed consecutively in clinical routine in an accredited laboratory by Cobas cholesterol/triglyceride/HDL reagents on Cobas c701 platforms. Blood pressure was measured, any history of CVD and reported tobacco use were recorded. Pain during the week preceding the assessment was self-assessed using a Visual Analogue Scale (VAS).

Aerobic capacity (VO_2_ max, ml/kg x min) was calculated based on age, sex, body weight, work load (watts) and working heart rate according to the submaximal test described by Åstrand [18] on a static bicycle (Monark Ergomedic 818E) while wearing a chest heart rate monitor (Polar Oy, Finland). All but two patients were tested for aerobic capacity at the time of diagnosis; those two patients undertook their first test six months later. Comparison of aerobic capacity in the whole group at baseline and at six months did not reveal any difference (p = 0.609), hence those two were included in the analyses. Since the age correction according to Åstrand [18] has been shown to underestimate the test value for patients with RA by approximately 10% [19], we also used the age correction equation for maximal heart rate according to Tanaka *et al*. [20] in order to increase the validity of the test. The test values are presented with the age correction according to Tanaka *et al*. [20] when not described otherwise. Functional ability was self-reported using the Swedish version of the Stanford Health Assessment Questionnaire (HAQ) [21].

According to routine in the early arthritis clinic, the patients answered the Swedish version of the questionnaire Arthritis Self-Efficacy Scale (ASES-S) [14] three months after diagnosis. The ASES-S scale comprises a total of 20 items, divided into three parts, concerning pain, function and other symptoms related to RA such as fatigue and depression. The scores are expressed as a value between 10 and 100 where a score of 10 represents the lowest possible self-efficacy level [14].

### Follow-up measures

The participating patients attended a follow-up clinic in 2015 when the average intervening period was 16.2 (range 13.0-19.2) years after being diagnosed with RA. All data concerning each individual were collected within a period of three months. Current medication was noted. Disease activity, pain, aerobic capacity, functional ability and self-efficacy were measured as described above. Aerobic capacity was estimated using an ergometer cycle (Monark 928G3) and chest worn heart rate monitor (Garmin Nordic Sweden AB). The patients were instructed not to exercise during the 24 hours prior to the visit, and to avoid heavy meals and tobacco for at least two hours before the visit. Every minute during the test the patients described their level of exertion according to the Borg scale of perceived exertion [22], and the test was interrupted with an assessment of 17 (very hard exertion level). All tests for aerobic capacity were conducted by the same physiotherapist (KH). The test values were compared to Swedish reference data [23].

The PWA was undertaken transcutaneously using an Arteriograph Type TL2 v. 3.0.0.3 (TensioMed^TM^ Ltd, Budapest, Hungary) showing measures not only of systolic (SBP), diastolic (DBP) and central blood pressure (SBPao), but also measures of pulse wave velocity (PWV) and augmentation index (AIx%). The PWV and AIx% are measures of arterial stiffness [24–26]. The patients rested supine in a quiet room for 10 minutes before undergoing three measurements in the right arm and the average values noted [27]. Ultrasonography was used to measure the carotid intima media thickness (IMT) while the patient was lying supine, using a Seqoia 512 ultrasound system [Siemens (Acuson) Corp, Upplands-Väsby, Sweden] with a 9L transducer [28].

Dual-energy X-ray absorbtiometry (DXA) was used to measure body composition according to the procedures recommended by the manufacturer [29] on a Lunar Prodigy X-ray Tube Housing Assembly, Brand BX-1L, Model 8743; GE Medical Systems, Madison, WI, USA). Body mass index (BMI) was calculated by dividing weight (kilograms) by the height (meters squared) [30]. Waist circumference was measured with the patient standing and the arms hanging freely with a measuring tape placed midway between the lower costal margin and the iliac crest. The measurement was made to the nearest 5 mm and performed at the end of a normal expiration [30]

## Statistics

Descriptive statistics are presented as median with inter-quartile range (Q1-Q3), mean with standard deviation (SD) or number with percentage, as appropriate. Differences between groups were analysed using Mann-Whitney U-test, and changes over time using Wilcoxon signed ranks test, or paired t-test. Associations between aerobic capacity and other variables were analysed with Spearman´s rank correlation test or simple linear regression analysis. In the multiple regression modeling, variables with a p-value ≤0.2 were further analyzed to establish the best fitting models depicting predictors for aerobic capacity at follow up.

A patient was defined as a responder to therapy according to the European League Against Rheumatism (EULAR) response criteria for RA, evaluated at 24 months after diagnosis [31]. Patients with no or a moderate response were considered non-responders and were, therefore, compared to patients with a good response. Patients were dichotomized into a younger and an older group at the median age for the whole group (40 years at baseline), and groups with lower and higher aerobic capacity at the median value (33.2 ml/kg/min). Missing data at baseline are considered to be random. The level of significance was set at a p-value of <0.05.

## Results

Descriptive data are presented in Table 1.

**Table 1:** Descriptive data of 25 patients with RA at baseline and at follow-up. Data are presented as median with inter-quartile range (Q1-Q3), mean with standard deviation (SD) or number (%) when appropriate. In comparisons over time, the p-value refers to Wilcoxon signed ranks test or t-test.

		Baseline	Follow-up	p
**Female/male**		20/5	20/5	
**Age, years**		40 (34.5-49.5)	58.0 (52.0-63.5)	
	Women, years	37.5 (34.0-47.8)	54.0 (49.8-62.0)	
	Men, years	48.0 (39.5-51-5)	61.0 (59.0-68.5)	
**Disease duration, years**^§^		0.1 (0.0)	16.2 (2.4)	
**ACPA pos, number (%)**		18 (72)		
**RF pos, number (%)**		24 (96)		
**Disease activity**				
	DAS28	4.73 (1.22)	2.93 (1.35)	.000
	DAS28, 12 months	2.94 (1.33)		
	DAS28, 24 months	3.07 (1.58)		
	AUC DAS28, 0-24 months	78.34 (26.21)		
	CRP, mg/L	11.0 (10.0-30.5)	1.8 (0.9-5.0)	.000
	ESR, mm/h	19.0 (13.5-37.5)	12.0 (4.0-21.0)	.028
	Tender joints, number	6.0 (1.5-11.0)	1.0 (0.0-2.5)	.000
	Swollen joints, number	9.0 (3.5-12.0)	2.0 (0.5-4.0)	.001
	Pain, VAS, (0-100 cm)	47.0 (24.2-56.8)	18.0 (7.0-60.5)	.003
Responder at 24 months, number (%)		12 (48)		
Non-responder at 24 months, number (%)		13 (52)		
**HAQ, (0-3), n = 24**			0.5 (0.25-0.97)	0.13 (0.0-0.32)	.002
**ASES-S**					
	Pain, (10-100) n = 15	64.0 (36.0-74.0)	66.0 (48.0-84.0)	.443
	Function, (10-100) n = 16	99.0 (70.0-100.0)	95.6 (88.4-98.9)	.308
	Other symptoms, (10-100) n = 15	78.3 (51.6-88.3)	78.3 (67.5-90.0)	.198
	Total, (10-100) n = 14	77.9 (56.8-85.3)	80.9 (67.6-88.1)	.433
**Aerobic capacity, ml O**_**2**_/kg x min*		32.3 (27.9-42.1)	33.2 (28.4-38.9)	.443
	Women, n = 20*	32.7 (29.4-42.7)	34.1 (27.6-41.2)	
	Men, n = 5*	27.7 (19.6-35.6)	31.6 (29.0-34.0)	
Aerobic capacity, L/min*		2.17 (1.9-2.5)	2.34 (2.1-2.6)	.253
	Women, n = 20*	2.17 (1.90-2.49)	2.29 (1.93-2.54)	
	Men, n = 5*	1.96 (1.87-2.75)	2.52 (2.39-3.44)	
**Aerobic capacity, ml/kg/min**^#^		32.0 (28.0-43.5)	31.0(27.0-37.3)	.089
	Women, n = 20^#^	34.0 (30.0-44.7)	32.0 (26.9-37.8)	
	Men, n = 5^#^	27.0 (23.0-35.0)	31.0 (28.0-37.5)	

ACPA = Anti citrullinated protein antibodies, RF = Rheumatoid factor, DAS28 = Disease Activity Score, AUC DAS28 = Area under the curve

DAS28, CRP = C-reactive proteins, ESR = Erythrocyte sedimentation rate, HAQ = Health Assessment Questionnaire, ASES-S = Arthritis

Self-Efficacy Scale.

* age correction according to Tanaka et al.

^#^ age correction according to Åstrand et al.

^§^ figure at baseline denotes time from symptom onset.

The mean (SD) disease duration at follow-up was 16.2 (2.4) years. According to the EULAR response criteria, twelve (48%) patients had a good response, seven (28%) a moderate response and six (24%) no response to therapy 24 months after diagnosis. At follow-up, all variables reflecting disease activity (DAS28) and functional ability (HAQ) had improved (Table 1).

At baseline, no patient had a history of a cardiovascular event, defined as myocardial infarction or stroke. Nine patients were smokers, one was being treated for hypertension and two for hyperlipidemia. The mean (SD) systolic blood pressure (measured manually) was 126.0 (14.6) and diastolic blood pressure was 76.4 (7.4) mmHg. The mean (SD) BMI was 23.4 (3.3) (kg/m^2^, n = 16) and the median (IQR) body weight was 64.0 (55.5-74.5) kg.

At follow-up, one patient had had a stroke, two patients were smokers, eleven were treated for hypertension and three for hyperlipidaemia. Twenty patients were being treated with synthetic DMARDs, nine with biological drugs, of whom eight received a combination of the two. Eleven were prescribed NSAIDs and four oral corticosteroids. Table 2 shows the follow-up parameters reflecting cardiovascular risk.

**Table 2:** Measures of cardiovascular risk factors at follow-up for the whole group (n = 25) and separately for patients with high and low aerobic capacity. Data are presented as median with inter-quartile range (Q1-Q3). In comparison between groups, the p-value refers to Mann-Whitney U-test.

		Whole group	Low aerobic capacity ≤ 33 ml/kg/min (n = 12)	High aerobic capacity >33 ml/kg/min (n = 13)	p
**Lipids**					
	Cholesterol, mmol/L	5.2 (4.8-5.8)	5.4 (5.1-6.1)	5.0 (4.8-5.8)	.378
	Triglycerides, mmol/L	1.0 (0.7-1.2)	1.0 (0.7-1.2)	1.0 (0.7-1.3)	.713
	HDL, mmol/L	1.6 (1.2-2.0)	1.6 (1.5-2.0)	1.6 (1.0-2.3)	.932
	LDL, mmol/L	3.2 (2.7-3.8)	3.4 (2.8-4.1)	3.2 (2.7-3.6)	.671
**Glucose, mmol/L**		5.4 (5.2-5.7)	5.6 (5.3-5.7)	5.2 (5.0-5.4)	.019
**Creatinine, mmol/L**		70.0 (60.8-75.0)	69.5 (59.0-74.5)	70.0 (63.5-76.5)	.713
**DXA**					
	BMI, kg/m^2^	26.5 (21.8-30.3)	28.2 (24.8-31.0)	22.5 (20.9-28.4)	.052
	Body weight, kg	72.3 (61.2-83.2)	79.0 (66.5-84.0)	66.6 (57.2-75.8)	.123
	Body fat,%	38.0 (30.6-46.2)	41.2 (33.3-48.2)	34.6 (29.2-42.9)	.123
	Android fat,%	43.7 (37.2-52.8)	51.2 (41.8-55.0)	38.0 (30.4-49.2)	.087
	Gynoid fat,%	46.0 (37.6-47.4)	46.0 (37.5-51.9)	42.8 (37.0-47.2)	.611
	Fat free weight, kg	43.4 (38.5-47.2)	42.9 (39.1-54.3)	43.4 (37.9-47.2)	.769
**Waist circumference, cm**		95.0 (76.5-101.5)	99.0 (84.9-106.0)	82.0 (75.2-99.5)	.123
	Women	83.2 (75.1-101.0)	97.0 (78.2-105.0)	80.0 (75.0-98.0)	
	Men	102.0 (95.0-115.0)			
**Pulse Wave Analysis**					
	SBP, mm Hg	131.3 (121.5-145.0)	134.5 (125.7-150.3)	125.7 (113.3-138.5)	.110
	DBP, mm Hg	80.3 (72.2-83.3)	81.0 (76.2-86.7)	80.3 (64.3-81.3)	.247
	PP bra, mm Hg	51.0 (45.6-57.7)	56.5 (45.5-63.7)	49.3 (46.3-53.0)	.137
	PP ao, mm Hg	50.7 (46.0-61.9)	56.0 (49.5-67.5)	47.7 (38.4-56.8)	.022
	SBP ao, mm Hg	129.9 (120.6-146.8)	136.2 (127.3-157.9)	128.0 (106.0-136.4)	.068
	RHR, beats/min	64.0 (59.8-68.7)	66.0 (61.5-68.8)	62.3 (58.3-71.5)	.376
	PWV ao, m/s	9.0 (7.7-11.0)	9.2 (8.6-11.8)	8.4 (7.4-10.6)	.137
	AIx bra,%	6.6 (-28.5-25.0)	8.2 (-5.7-32.1)	-27.6 (-38.6-23.3)	.087
	AIx ao,%	41.0 (23.2-50.3)	41.8 (29.6-53.9)	23.7 (18.1-49.4)	.098
**Carotid ultrasound**					
	Mean cIMT, mm	0.65 (0.58-0.78)	0.66 (0.60-0.80)	0.64 (0.57-0.72)	.214

HDL = High density lipids, LDL = low density lipids, DXA = Dual-energy X-ray absorbtiometry, BMI = Body mass index, SBP = Systolic blood pressure, DBP = Diastolic blood pressure, PP bra = Brachial Pulse pressure, PP ao = Aortic Pulse pressure, SBP ao = Aortic systoloc blood pressure, RHR = Resting heart rate, PWV = Pulse wave velocity, Aix bra = Brachial Augmentation index, Aix ao = Aortic Augmentation index, cIMT = Carotid intima media thickness.

### Aerobic capacity at disease onset and at follow-up

Compared with Swedish reference values for different age groups and sexes, the aerobic capacity for both men and women was below average at baseline and above average at follow-up (Table 1) [23]. For the whole group, the test value did not change significantly over time. The test value based on the age correction according to Åstrand [18] showed a slight decrease at follow-up, although not to a statistically significant level. Women had higher values than men at both time points, but the median age for men was higher compared with women (48.0 vs 37.5 years at baseline) (Table 1). Individual changes of aerobic capacity (ml/kg/min) from baseline to follow-up are presented in supplementary S1 Fig.

**Fig 2:**
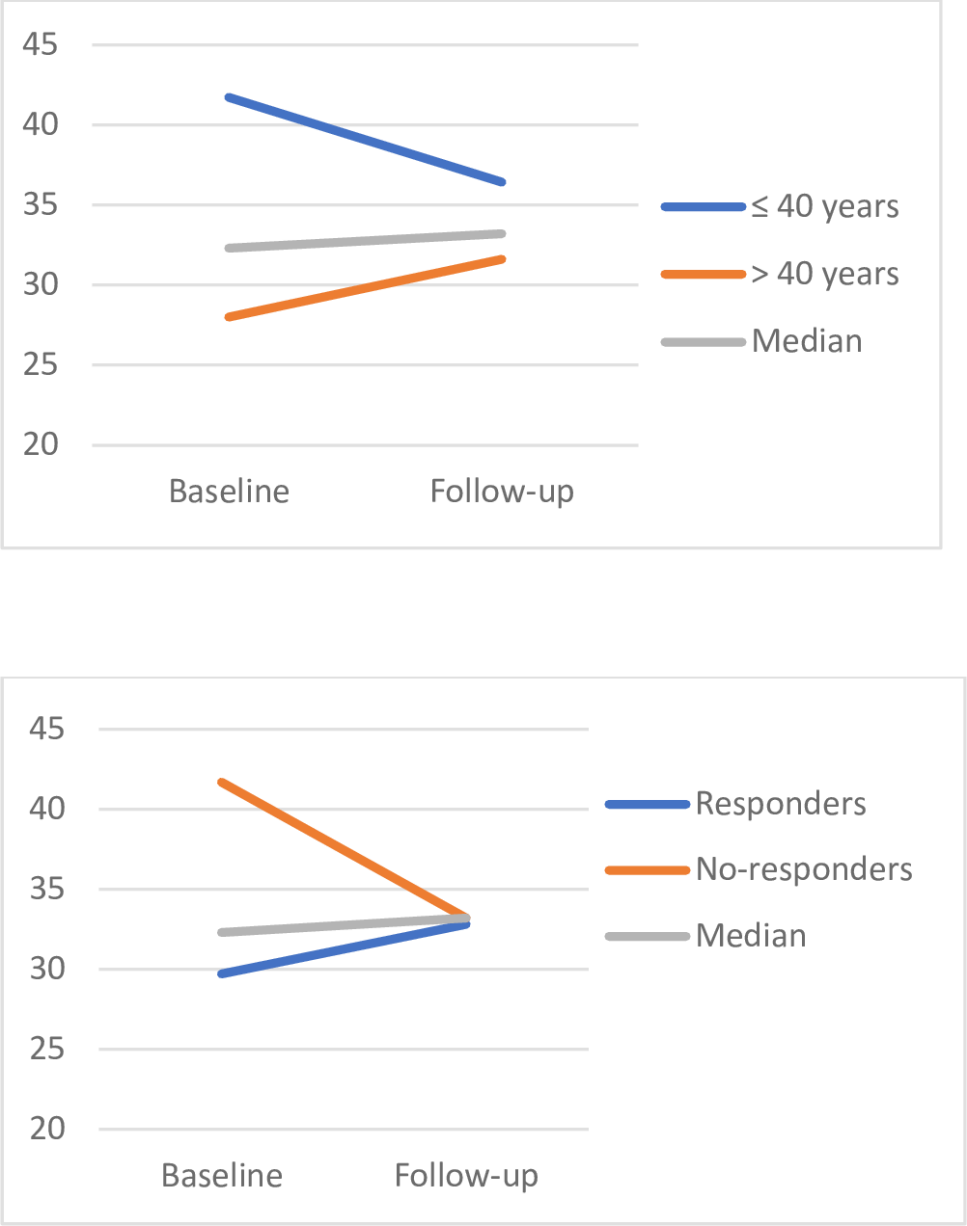
Change of aerobic capacity (ml/kg/min) from baseline to follow-up for younger and older patients and for responders and non-responders to therapy. The values are presented as medians.

Dichotomizing the whole group into a younger and an older group at the median age (40 years) shows a median test value of [ml/kg/min (IQR)] 41.7 (32.4-47.9) at baseline and 36.4 (27.9-44.2) at follow-up for the younger group and 28.0 (26.4-32.0) at baseline and 31.6 (28.4-34.1) at follow-up for the older group (Fig 2). Descriptive data separate for younger and older patients are presented in S1 Table.

Numerically, the aerobic capacity deteriorated in the younger but improved in the older patients, and there was a statistically significant difference between younger and older patients at baseline, but not at the time of follow-up (p = 0.002 and p = 0.103 respectively). Functional ability, as measured by HAQ, was significantly lower in the older patients at baseline (p = 0.023), but no other variable representing disease activity, joint- and muscle function or functional ability differed significantly at baseline or at follow-up.

When dichotomizing the patients according to response (n = 12) and no response (n = 13) to therapy at 24 months, the median [ml/kg/min (IQR)] value for the responders was 29.7 (27.3-35.4) at baseline and 32.8 (27.4-34.6) at follow-up. The corresponding values for the non-responders were 41.7 (29.1-45.9) and 33.2 (29.7-41.4) respectively (Fig 2). However, there were no statistically significant changes over time or differences between the groups. Descriptive data separate for responders and non-responders are presented in S2 Table.

### Factors at follow-up related to aerobic capacity at follow-up

Table 3 presents the results from the correlation analyses between variables at follow-up and aerobic capacity at the same time point.

**Table 3:** Correlation coefficients (r_s_) for aerobic capacity (ml/kg/min) at follow-up in relation to other variables at follow-up. The correlations were calculated using the Spearman rank correlation coefficient method.

Variables at follow-up		Aerobic capacity at follow-up	
		r_s_	p
**Age, years**		-.381	.060
**DAS28**		.100	.643
**Glucose, mmol/L**		-.508	.016
**DXA**			
	BMI, kg/cm^2^	-.434	.030
	Body weight, kg	-.387	.056
	Body fat,%	-.419	.037
	Android fat,%	-.379	.061
	Fat Free Weight, kg	-.053	.801
**Waist circumference, cm**		-.378	.063
**Pulse Wave Analysis**			
	SBP, mm Hg	-.335	.101
	DBP, mm Hg	-.267	.198
	PP bra, mm Hg	-.276	.181
	PP ao, mm Hg	-.405	.044
	SBP ao, mm Hg	-.343	.093
	RHR, beats/min	-.424	.034
	PWV, m/s	-.343	.093
	AIx bra,%	-.283	.170
	AIx ao,%	-.271	.190
**ASES-S function**		.464	.020

DAS28 = Disease Activity Score, DXA = Dual-energy X-ray absorbtiometry, BMI = Body mass index, SBP = Systolic blood pressure, DBP = Diastolic blood pressure, PP bra = Brachial Pulse pressure, PP ao = Aortic Pulse pressure, SBP ao = Aortic Systolic blood pressure, RHR = Resting heart rate, PWV = Pulse wave velocity, Aix bra = Brachial Augmentation index, Aix ao = Aortic Augmentation index, ASES-S function = Arthritis Self-Efficacy Scale subscale for performing different tasks.

Patients with higher aerobic capacity had lower follow-up levels of blood glucose, BMI, body fat, aortic pulse pressure and resting heart rate and also higher self-efficacy for performing different tasks.

### Associations between baseline aerobic capacity and future measures of cardiovascular risk factors and atherosclerosis

It was found that higher baseline aerobic capacity was significantly associated with higher aerobic capacity at follow-up, and to more favorable values at follow-up in terms of BMI, body weight, waist circumference, brachial pulse pressure and self-efficacy for performing different tasks (Table 4).

**Table 4:** Correlation coefficients (r_s_) for aerobic capacity (ml/kg/min) at baseline in relation to other variables at follow-up. The correlations were calculated using the Spearman rank correlation coefficient method.

Variables at follow-up		Aerobic capacity at baseline	
		r_s_	p
**Age, years**		-.469	.018
**CRP, mg/L**		-.373	.072
**Glucose, mmol/L**		-.392	.071
**Aerobic capacity, ml/kg/min**		.557	.004
**DXA**			
	BMI, kg/cm^2^	-.401	.047
	Body weight, kg	-.409	.043
	Body fat,%	-.140	.506
	Android fat,%	-.222	.287
**Waist circumference, cm**		-.498	.011
**Pulse Wave Analysis**			
	SBP, mm Hg	-.385	.057
	DBP, mm Hg	-.295	.153
	PP bra, mm Hg	-.415	.039
	PP ao, mm Hg	-.286	.166
	SBP ao, mm Hg	-.352	.084
	RHR, beats/min	-.379	.062
	PWV, m/s	-.370	.069
**ASES-S function**		.420	.037

CRP = C-reactive protein, DXA = Dual-energy X-ray absorbtiometry, BMI = Body mass index, SBP = Systolic blood pressure, DBP = Diastolic blood pressure, PP bra = Brachial Pulse pressure, PP ao = Aortic Pulse pressure, SBP ao = Aortic Systolic blood pressure, RHR = Resting heart rate, PWV = Pulse wave velocity, ASES-S function = Arthritis Self-Efficacy Scale subscale for performing different tasks.

### Baseline factors predicting aerobic capacity after 16 years

Table 5 presents the relationship between baseline variables and aerobic capacity at follow-up tested with simple regression analyses. The level of aerobic capacity at follow-up was predicted by DAS28 at inclusion, DAS28 at 24 months, AUC DAS28 0-24 months and baseline aerobic capacity. Also, when analysing aerobic capacity as L/min, the same associations were found; DAS28 at inclusion (p = 0.017), DAS28 at 24 months (p = 0.005), AUC DAS28 0-24 months (p = 0.039) and baseline aerobic capacity as L/min (p = 0.044).

**Table 5:** Simple linear regression coefficients for variables at baseline associated with aerobic capacity (ml/kg/min) at follow-up.

Variable at baseline	Aerobic capacity at follow-up		
	β	95% CI	p
Sex	2.45	-5.78-10.68	.545
Age, years	-.28	-.61-.04	.084
DAS28, inclusion	-3.01	-5.46–-.55	.019
DAS28, 24 months	-2.09	-4.03–-.15	.036
AUC DAS28, 0-24 months	-.15	-.26–-.03	.014
Aerobic capacity, ml/kg/min	.45	.16-.75	.004
Body weight, kg	-.18	-.42-.07	.152
Ever smoker	-4.25	-10.63-2.14	.182
HAQ	-4.86	-12.25-2.52	.186

AUC DAS28 = Area under the curve for Disease Activity Score, HAQ = Health Assessment Questionnaire.

In the next step, multiple linear regression analyses were performed to find the model best explaining the variation in aerobic capacity at follow-up. Disease activity during the first 24 months after diagnosis (AUC DAS28) explained 53% of the aerobic capacity level at follow-up in a model including baseline aerobic capacity (Table 6). When the DAS value at baseline or after 24 months were tested, results were pointing in the same direction (S3 Table).

**Table 6:** Multiple linear regression model. Variables in early disease associated with aerobic capacity (ml/kg/min) at follow-up.

	β	CI 95%	R^2^	p
**Aerobic capacity baseline**	.44	.20-.69	.53	.001
**AUC DAS28 0-24 months**	-.14	-.23–-.05	.53	.004

DAS = Disease Activity Score, AUC DAS28 = Area under the curve for Disease Activity Score.

The impact of self-efficacy was evaluated in addition to baseline aerobic capacity and disease activity during the first 24 months after diagnosis. This model included only 14 patients, but explained 71% of the variation in aerobic capacity levels at follow-up (Table 7).

**Table 7:** Multiple linear regression model. Variables in early disease associated with aerobic capacity (ml/kg/min) at follow-up.

	β	CI 95%	R^2^	p
**Aerobic capacity baseline**	.49	.18-.80	.71	.006
**AUC DAS28** **0-24 months**	-.14	-.23–-.04	.71	.009
**ASES-S** **baseline**	.03	-.12-.18	.71	.626

AUC DAS28 = Area under the curve for Disease Activity Score, ASES-S = Arthritis Self-Efficacy Scale, total score

Thirty-two patients undertook tests for aerobic capacity at baseline but did not take part in the follow-up (Fig 1). The 32 non-participants did not, however, differ significantly from the participants in terms of sex, age, disease activity, functional ability and level of aerobic capacity at baseline.

## Discussion

In this prospective study, aerobic capacity was maintained over time. Compared with Swedish reference data [23], the test value was below average at disease onset and above average after 16 years. At follow-up, aerobic capacity was significantly associated with CV risk factors and atherosclerosis. Also, baseline aerobic capacity could predict future BMI, body weight, waist circumference, and pulse pressure, which may contribute to CV risk. Furthermore, higher disease activity in early disease predicted a lower aerobic capacity at follow-up.

To the best of our knowledge this is the first prospective study presenting the change of aerobic capacity over a longer period of time in patients with RA. Cross-sectional studies within the field have presented inconsistent results. Patients with RA have shown significantly reduced aerobic capacity in comparison with healthy controls [32, 33], and with reference data accumulated from the general population [34]. Metsios *et al*. [8] concluded that the aerobic capacity was alarmingly low [mean (SD) 20.9 (5.7) ml/kg/min] in 144 RA patients with disease lasting six years and low disease activity according to DAS28 score. On the contrary, other studies have presented no difference between patients with RA compared with healthy controls [35, 36], or compared with reference data from the general population [37, 38].

The patients in this study were able to maintain their aerobic capacity over time. Apparently, those with an active life style could manage to keep up with their exercise habits, despite the consequences of RA. The history of exercising has been shown to predict future exercise status in patients with RA [39, 40]. The multidisciplinary routine program, with an emphasize on physical activity, may also have facilitated the patients’ maintenance or improvement of exercise behavior over time.

Younger patients had significantly higher aerobic capacity compared with the older group at baseline, but not at the time of follow-up, indicating a decline in younger patients. This is in line with their functional ability, which was significantly better in younger patients at baseline, but the difference was no longer apparent at follow-up. The younger patients may have been physically active in more vigorous activities at baseline, but may have, due to emerging activity limitations, but also joint symptoms, fatigue and fear of aggravating symptoms, changed their activity patterns to lighter activities over time. In this regard, Munsterman and colleagues [34] concluded in their meta-analysis that patients with RA, in general, spend more time in light and moderate activities and less in vigorous activities compared with control subjects.

Aerobic capacity is known to be a strong, independent predictor for CVD and all-cause mortality in the general population [6]. In the present study, follow-up levels of aerobic capacity were related to CV risk factors, *i*.*e*. blood glucose levels, BMI, body fat, resting heart rate and aortic pulse pressure, at the same time point, consistent with a previous report on patients with RA [8]. However, aerobic capacity at disease onset also predicted CV risk factors, *i*.*e*. BMI, body weight, waist circumference and pulse pressure, after 16 years. Thus, our results suggest that aerobic capacity also has a predictive value of future CV risk factors in RA patients, and emphasize the importance of guiding patients with low aerobic capacity into changing their physical activity behavior.

There were several indications of a relationship between inflammatory status and the level of aerobic capacity. Higher disease activity during the first two years after diagnosis predicted lower aerobic capacity at follow-up, regardless of whether the test value was analysed as ml/kg/min or L/min. Correspondingly; responders to therapy numerically increased their aerobic capacity over time, while non-responders deteriorated (29.7 to 32.8 and 41.7 to 33.2 respectively). This association may be explained by pain, fatigue and impaired joint- and muscle function, decreasing self-efficacy and leading to avoidance of high intensity physical activities. Pharmacological treatment of RA is continuously becoming more efficient in reducing disease activity in patients with RA [41]. Consequently, in patients diagnosed recently, the impact of disease activity on aerobic capacity may be reduced accordingly.

In this analysis, several associations were found between self-efficacy and aerobic capacity. Higher baseline aerobic capacity correlated with higher follow-up levels of self-efficacy and higher baseline self-efficacy predicted higher aerobic capacity at follow-up. In addition, higher self-efficacy was associated with higher aerobic capacity at follow-up. These results elucidate the importance of self-efficacy for the maintenance of physical activity as described in the literature [15].

A maximal test is the “gold standard” for measuring aerobic capacity. A submaximal test was used because the average patient with RA may not be able to achieve a maximal effort due to disease-related pain and physical dysfunction. The Åstrand test has been considered highly valid and feasible [19], and has been recommended in physiotherapy guidelines for patients with RA [42]. In order to increase validity of the test, we used the age correction for predicting maximal heart rate as described by Tanaka *et al*. [20], but also presented the test values with the age correction according to Åstrand [18] to facilitate comparisons with other studies. The Swedish reference data on aerobic capacity [23] are based on age correction according to Åstrand, but the test values were below average at baseline and above average at follow-up, regardless the method of age correction.

A limitation of the present study is the small sample size and the consequent difficulties of generalizability. However, we included as many as possible of all patients diagnosed with RA in the county of Västerbotten during 1995-2002 (Fig 1). Furthermore, the included patients were comparable to the other patients in the same age group and diagnosed with RA in Västerbotten during this period. Studies with larger subject cohorts are warranted in order to explore further this topic.

To conclude, the present study expands our understanding of the aerobic capacity changes, predicting factors and the possible contribution of aerobic capacity level to risk factors for CVD in patients with RA. The aerobic capacity was maintained over 16 years and high disease activity predicted lower aerobic capacity at follow-up. The associations between aerobic capacity and several risk factors for CVD indicates, that aerobic capacity has a role to play in the management of CV risk in RA, stressing the importance of regular testing of aerobic capacity with a priority of patients with higher disease activity.

## Supporting information

S1 Fig Individual changes of aerobic capacity (ml/kg/min) from baseline to follow-up. (DOCX)

S1 Table Descriptive data of 25 RA patients at baseline and at follow-up, dichotomized into a younger (≤40 years) and older (>40 years) group. Data are presented as median with inter-quartile range (Q1-Q3), or number (%) as appropriate.
(DOCX)

S2 Table Descriptive data of 25 RA patients at baseline and at follow-up, dichotomized according to response to therapy 24 months after diagnose. Data are presented as median with inter-quartile range (Q1-Q3), or number (%) as appropriate.
(DOCX)

S3 Table Two multiple linear regression models. Variables in early disease associated with aerobic capacity (ml/kg/min) at follow-up.
(DOCX)
