# Supplementary material for: Aerobic capacity over 16 years in patients with rheumatoid arthritis: Relationship to disease activity and risk factors for cardiovascular disease
Source: PLoS One. 2017 Dec 22;12(12):e0190211. doi: 10.1371/journal.pone.0190211 (PMC5741242; doi:10.1371/journal.pone.0190211)
Supplement: S1 Fig — (DOCX) [file pone.0190211.s001.docx]

S1 Figure. Individual changes of aerobic capacity (ml/kg/min) from baseline to follow-up.
