# Supplementary material for: Aerobic capacity over 16 years in patients with rheumatoid arthritis: Relationship to disease activity and risk factors for cardiovascular disease
Source: PLoS One. 2017 Dec 22;12(12):e0190211. doi: 10.1371/journal.pone.0190211 (PMC5741242; doi:10.1371/journal.pone.0190211)
Supplement: S1 Table — Data are presented as median with inter-quartile range (Q1-Q3), or number (%) as appropriate. (DOCX) [file pone.0190211.s002.docx]

| **S1 Table. Descriptive data of 25 RA patients at baseline and at follow-up, dichotomized into a younger (≤40 years) and older (>40 years) group. Data are presented as median with inter-quartile range (Q1-Q3), or number (%) as appropriate.** | | | | | | | | | | | | |
| --- | --- | --- | --- | --- | --- | --- | --- | --- | --- | --- | --- | --- |
|  | | |  | | | | | | **Age ≤40 years at baseline** | | **Age >40 years at baseline** | |
|  | | |  | | | | | | **Baseline** | **Follow-up** | **Baseline** | **Follow-up** |
| **Female/male** | | |  | | | | | | 11/1 |  | 9/4 |  |
| **Age, years** | | |  | | | | | | 34.5 (23.2-37.0) | 52.0 (42.0-53.8) | 49.0 (40.0-51.5) | 63.0 (58.5-67.5) |
| **Disease duration, years**^§^ | | | | |  | | | | 0.8 (0.63-1.14) | 16.9 (14.3-18.3) | 1.0 (0.6-1.2) | 14.6 (13.5-18.7) |
| **ACPA pos, number (%)** | | | |  | | | | | 9 (75) |  | 9 (69) |  |
| **RF pos, number (%)** | | |  | | | | | | 11 (92) |  | 13 (100) |  |
| **Disease activity** | | |  | | | | | |  |  |  |  |
|  | DAS28 | | | | | | | | 4.5 (3.0-5.6) | 2.0 (1.4-4.3) | 5.0 (4.3-5.5) | 3.6 (2.2-4.1) |
|  | DAS28, 12 months | | | | | | | | 2.7 (1.6-4.3) |  | 2.6 (1.9-3.9) |  |
|  | DAS28, 24 months | | | | | | | | 2.7 (1.2-3.8) |  | 2.8 (2.1-4.8) |  |
|  | AUC DAS28, 0-24 months | | | | | | | | 76.2 (52.9-97.1) |  | 75.0 (63.2-93.7) |  |
|  | CRP, mg/L | | | | | | | | 10.0 (10.0-29.0) | 1.4 (0.7-6.8) | 15.0 (10.0-34.0) | 2.1 (1.0-4.5) |
|  | ESR, mm/h | | | | | | | | 15.0 (6.0-31.0) | 8.5 (4.0-21.0) | 26.0 (16.0-41.0) | 13.0 (12.0-24.8) |
|  | Tender joints, number | | | | | | | | 4.0 (1.2-14.0) | 0.0 (0.0-3.5) | 6.0 (1.5-9.5) | 1.0 (0.5-2.5) |
|  | Swollen joints, number | | | | | | | | 8.5 (2.0-12.5) | 1.5 (0.0-5.2) | 9.0 (5.0-13.5) | 3.0 (1.0-4.0) |
|  | Pain, VAS, (0-100 cm) | | | | | | | | 43.0 (16.0-52.0) | 13.0 (1.8-62.2) | 49.0 (33.5-65.0) | 18.0 (7.5-50.0) |
| Responder at 24 months, number (%) | | | | | | | | | 4 (33) |  | 8 (62) |  |
|  | Non-responder at 24 months, number (%) | | | | | | | | 8 (67) |  | 5 (38) |  |
| **HAQ, (0-3), n=24** | | |  | | | | | | 0.38 (0.13-0.50) | 0.19 (0.00-0.44) | 0.88 (0.50-1.00) | 0.13 (0.00-0.32) |
| **ASES-S** | | |  | | | | | |  |  |  |  |
|  | | Pain, (10-100) n=15 | | | | | | | 47.0 (36.5-81.0) | 69.5 (48.0-85.0) | 64.0 (26.0-74.0) | 66.0 (45.0-84.0) |
|  | | Function, (10-100) n=16 | | | | | | | 97.2 (79.8-100.0) | 95.6 (89.2-98.9) | 99.5 (65.2-100.0) | 95.6 (87.2-98.9) |
|  | | Other symptoms, (10-100) n=15 | | | | | | | 73.4 (52.9-90.8) | 76.7 (66.7-87.9) | 78.3 (43.3-88.3) | 86.7 (73.3-90.0) |
|  | | Total, (10-100) n=14 | | | | | | | 76.6 (61.1-85.4) | 82.0 (67.0-88.6) | 77.9 (44.4-86.4) | 80.3 (68.2-88.3) |
| **Aerobic capacity, ml O_2_/kg x min*** | | | | | | | |  | 41.7 (32.4-47.9) | 36.4 (27.9-44.2) | 28.0 (26.4-32.0) | 31.6 (28.4-34.1) |
| **Aerobic capacity, L/min*** | | | | | |  | | | 2.4 (1.9-3.2) | 2.4 (2.2-2.9) | 2.0 (1.9-2.2) | 2.3 (1.8-2.6) |
| **Aerobic capacity, ml/kg/min^#^** | | | | | | |  | | 42.5 (33.5-49.5) | 35.6 (29.6-41.6) | 28.0 (26.5-31.5) | 30.0 (26.4-32.0) |
| ACPA=Anti citrullinated protein antibodies, RF=Rheumatoid factor, DAS28=Disease Activity Score, AUC DAS28=Area under the curve  DAS28, CRP=C-reactive proteins, ESR=Erythrocyte sedimentation rate, HAQ=Health Assessment Questionnaire, ASES-S=Arthritis  Self-Efficacy Scale.  * age correction according to Tanaka et al.  ^#^ age correction according to Åstrand et al.  ^§^ figure at baseline denotes time from symptom onset. | | | | | | | | | | | | |
