# Supplementary material for: Aerobic capacity over 16 years in patients with rheumatoid arthritis: Relationship to disease activity and risk factors for cardiovascular disease
Source: PLoS One. 2017 Dec 22;12(12):e0190211. doi: 10.1371/journal.pone.0190211 (PMC5741242; doi:10.1371/journal.pone.0190211)
Supplement: S2 Table — Data are presented as median with inter-quartile range (Q1-Q3), or number (%) as appropriate. (DOCX) [file pone.0190211.s003.docx]

| **S2 Table. Descriptive data of 25 RA patients at baseline and at follow-up, dichotomized according to response to therapy 24 months after diagnose. Data are presented as median with inter-quartile range (Q1-Q3), or number (%) as appropriate.** | | | | | | | | | | | | |
| --- | --- | --- | --- | --- | --- | --- | --- | --- | --- | --- | --- | --- |
|  | |  | | | | | | **Responders at 24 months** | | **Non-responders at 24 months** | | |
|  | |  | | | | | | **Baseline** | **Follow-up** | | **Baseline** | **Follow-up** |
| **Female/male** | |  | | | | | | 9/3 |  | | 11/2 |  |
| **Age, years** | |  | | | | | | 40.0 (37.2-50.8) | 59.0 (54.2-66.5) | | 37.0 (29.0-46.0) | 54.0 (48.5-60.0) |
| **Disease duration, years**^§^ | | | |  | | | | 0.8 (0.6-1.14) | 17.4 (14.0-18.9) | | 1.0 (0.5-1.2) | 14.6 (13.5-18.0) |
| **ACPA pos, number (%)** | | |  | | | | | 8 (67) |  | | 10 (77) |  |
| **RF pos, number (%)** | |  | | | | | | 12 (100) |  | | 12 (92) |  |
| **Disease activity** | |  | | | | | |  |  | |  |  |
|  | DAS28 | | | | | | | 4.6 (4.2-5.4) | 3.4 (1.6-3.8) | | 5.0 (3.4-5.6) | 2.5 (1.9-4.3) |
|  | DAS28, 12 months | | | | | | | 2.3 (1.9-2.7) |  | | 3.7 (2.1-4.3) |  |
|  | DAS28, 24 months | | | | | | | 2.1 (1.6-2.7) |  | | 4.3 (2.9-5.2) |  |
|  | AUC DAS28, 0-24 months | | | | | | | 69.0(56.4-82.5) |  | | 85.6 (64.4-104.9) |  |
|  | CRP, mg/L | | | | | | | 11.5 (10.0-36.8) | 2.0 (0.9-5.0) | | 10.0 (10.0-30.5) | 1.8 (0.7-5.7) |
|  | ESR, mm/h | | | | | | | 18.5 (16.0-37.5) | 13.0 (5.5-26.2) | | 19.0 (6.5-44.5) | 11.0 (4.0-14.5) |
|  | Tender joints, number | | | | | | | 6.0 (2.0-10.2) | 1.0 (0.0-1.8) | | 4.0 (1.0-13.5) | 1.0 (0-5.5) |
|  | Swollen joints, number | | | | | | | 8.5 (6.0-10.8) | 1.0 (0.2-3.0) | | 10.0 (2.5-16.0) | 3.0 (0.5-5.5) |
|  | Pain, VAS, (0-100 cm) | | | | | | | 46.0 (17.0-67.0) | 15 (7.0-72.2) | | 48.0 (26.0-53.5) | 18.0 (6.5-44.0) |
| **HAQ, (0-3), n=24** | |  | | | | | | 0.75 (0.50-1.00) | 0.13 (0.00-0.25) | | 0.38 (0.13-0.82) | 0.13 (0.00-0.44) |
| **ASES-S** | |  | | | | | |  |  | |  |  |
|  | Pain, (10-100) n=15 | | | | | | | 54.0 (29.0-71.5) | 67.0 (48.5-85.0) | | 64.0 (36.0-84.0) | 62.0 (45.0-84.0) |
|  | Function, (10-100) n=16 | | | | | | | 95.6 (66.1-100.0) | 95.6 (88.3-100.0) | | 99.0 (88.9-100.0) | 95.6 (87.2-98.4) |
|  | Other symptoms, (10-100) n=15 | | | | | | | 78.4 (34.6-88.3) | 78.3 (67.1-87.9) | | 78.3 (56.7-91.7) | 86.7 (70.8-93.1) |
|  | Total, (10-100) n=14 | | | | | | | 81.5 (40.7-85.2) | 82.1 (67.9-88.8) | | 75.0 (63.0-93.0) | 80.9 (66.8-88.4) |
| **Aerobic capacity, ml O_2_/kg x min*** | | | | | | |  | 29.7 (27.3-35.4) | 32.8 (27.4-34.6) | | 41.7 (29.1-45.9) | 33.2 (29.7-41.4) |
| **Aerobic capacity, L/min*** | | | | |  | | | 2.1 (1.9-2.3) | 2.3 (2.1-2.9) | | 2.2 (1.9-2.7) | 2.3 (1.9-2.5) |
| **Aerobic capacity, ml/kg/min^#^** | | | | | |  | | 30.0 (26.5-35.8) | 30.0 (26.3-33.8) | | 42.0 (30.0-45.0) | 34.2 (27.0-39.2) |
| ACPA=Anti citrullinated protein antibodies, RF=Rheumatoid factor, DAS28=Disease Activity Score, AUC DAS28=Area under the curve  DAS28, CRP=C-reactive proteins, ESR=Erythrocyte sedimentation rate, HAQ=Health Assessment Questionnaire, ASES-S=Arthritis  Self-Efficacy Scale.  * age correction according to Tanaka et al.  ^#^ age correction according to Åstrand et al.  ^§^ figure at baseline denotes time from symptom onset. | | | | | | | | | | | | |
