# Supplementary material for: Aerobic capacity over 16 years in patients with rheumatoid arthritis: Relationship to disease activity and risk factors for cardiovascular disease
Source: PLoS One. 2017 Dec 22;12(12):e0190211. doi: 10.1371/journal.pone.0190211 (PMC5741242; doi:10.1371/journal.pone.0190211)
Supplement: S3 Table — Variables in early disease associated with aerobic capacity (ml/kg/min) at follow-up. (DOCX) [file pone.0190211.s004.docx]

| **S3 Table. Two multiple linear regression models. Variables in early disease associated with aerobic capacity (ml/kg/min) at follow-up.** | | | | | | | | |
| --- | --- | --- | --- | --- | --- | --- | --- | --- |
|  | **Model 1** | | | | **Model 2** | | | |
|  | **β** | **CI 95%** | **R^2^** | **p** | **β** | **CI 95%** | **R^2^** | **p** |
| **Aerobic capacity baseline** | .37 | .07-.66 | .40 | .018 | .44 | .17-.70 | .46 | .003 |
| **DAS28 baseline** | -2.09 | -4.43-0.25 | .40 | .077 | - | - | - | - |
| **DAS28**  **24 months** | - | - | - | - | -1.96 | -3.58--0.35 | .46 | .020 |
| DAS=Disease Activity Score | | | | | | | | |
